# Supplementary material for: A novel dual-branch network for comprehensive spatiotemporal information integration for EEG-based epileptic seizure detection
Source: PLoS One. 2025 Jun 26;20(6):e0321942. doi: 10.1371/journal.pone.0321942 (PMC12200712; doi:10.1371/journal.pone.0321942)
Supplement: Attachment — Submitted filename: Comment to author(s).pdf [file pone.0321942.s001.pdf]

Comment to author(s):

I have gone through this article. It is based on epileptic seizure detection by employing a DeepWalk algorithm on EEG datasets. The overall study has great potential to add significant value to relevant fields. However, some points should be considered before the final acceptance of this article.

1. The evaluation scores were significantly high with an accuracy of 99.54%. Why does it not reach 100%, and how can overfitting be understood in the proposed work? It is possible to add a description related to these concerns to the Discussion section, or wherever possible in the manuscript, it is feasible.
2. Please mention the acronyms in full form when they are first used in the manuscript, such as "GMM," on page 9.”
3. Please check Page no. 5, equation 2 and equation 3, the variables "bk" and "Dt" are defined somewhere or not. Also, check all the typos if they are available in the manuscript, such as in page no. 8, section 4.3.1, "...proposed model dem onstrated..."

Overall, the manuscript has great potential and the proposed work is highly valuable. We look forward to getting this article to the next stage of article processing and acceptance.
