# Supplementary material for: A novel dual-branch network for comprehensive spatiotemporal information integration for EEG-based epileptic seizure detection
Source: PLoS One. 2025 Jun 26;20(6):e0321942. doi: 10.1371/journal.pone.0321942 (PMC12200712; doi:10.1371/journal.pone.0321942)
Supplement: Attachment — Submitted filename: Response to Reviewers.pdf [file pone.0321942.s002.pdf]

Response to Reviewers

Dear Editor and Reviewers:

Thank you for your letter and the reviewers' comments concerning our manuscript entitled "A Novel Dual-Branch Network for Comprehensive Spatiotemporal Information Integration for EEG-based Epileptic Seizure Detection (No. PONE-D-24-53519)". We deeply appreciate the constructive comments, helpful critiques, and favorable assessments from all reviewers. Those comments are valuable and have significantly contributed to the revision and improvement of our paper. We have carefully reviewed the comments and made the necessary modifications to the resubmitted manuscript. Below are our detailed point-by-point responses to each comment.

Editor's Comments:

Thank you for your thorough review of our manuscript, bring your constructive feedback and positive comments on the proposed Deepwalk-TS algorithm for epilepsy detection. Below, we address the issues you raised and outline the changes made to improve the paper's quality and suitability for publication.

1. Please discuss whether the application of the algorithm is possible for online detection (i.e., close loop system).

We have discussed the online application of Dual-Branch Deepwalk Transformer Spatiotemporal Fusion Network (Deepwalk-TS) in detail in the "Discussion" section. It can be determined that method shows great application potential in online epilepsy detection. The algorithm innovatively integrates the spatial information analysis of Deepwalk-based and the temporal feature extraction of Guided-CNN Transformer, dynamically and efficiently processes EEGs, and has achieved accurate and timely epilepsy detection. Although the real-time performance and feedback integration need to be further optimized, the proposed method provides a solid foundation for advanced clinical systems to support online epilepsy detection in a timely manner.

For real-time detection, the spatiotemporal information integration enables the algorithm to process continuous EEGs effectively, achieving highly accurate seizure detection. The Deepwalk-based graph construction dynamically captures spatial relationships across EEG channels, while the Transformer module efficiently processes time-series data, ensuring real-time detection capability. Although current implementations may require minor delays for feature extraction, the framework can be optimized for low-latency detection through techniques such as **model pruning, lightweight architecture adaptations, and parallel computations**. The model is well-suited for online streaming data, making it feasible for real-time monitoring.

Regarding integration into closed-loop systems, our study primarily focuses on algorithmic design and localized detection using multiple datasets. The method's

robust ability to analyze spatiotemporal EEG features ensures reliable seizure detection, even in complex clinical scenarios. While the method emphasizes accuracy and strong performance in static evaluations, further optimization is required to enhance real-time performance and feedback integration. As part of a feedback loop, the system can process real-time EEG data streams to continuously update the prediction model, **leveraging adaptive learning techniques such as incremental training or online fine-tuning.** Additionally, **incorporating active learning or adaptive model** updates based on real-time feedback could further improve the model's ability to adapt to new seizure patterns. The proposed framework promises seamless integration into clinical workflows, enabling early intervention and real-time feedback in closed-loop systems for epilepsy management.

2. What are the strengths and weaknesses of the proposed framework? Please include them in the paper.

We have added a detailed discussion of the strengths and weaknesses of our method in the revised manuscript to enhance the clarity and depth of the paper.

The Deepwalk-TS has **several notable strengths:** First, its dual-branch architecture effectively integrates spatial and temporal features, addressing key challenges in EEGs analysis. The Deepwalk-based spatial branch dynamically captures high-order relationships among EEG channels through adaptive graph construction, ensuring that spatial dependencies are preserved and utilized efficiently. Second, the Guided-CNN Transformer temporal branch leverages CNN’s spatial feature extraction capabilities and the Transformer’s global attention mechanism to model temporal dependencies effectively. This combination enables the model to extract meaningful sequential patterns, significantly enhancing its sensitivity to seizure-related signals. Third, extensive experiments demonstrate that Deepwalk-TS achieves state-of-the-art performance on multiple datasets, underscoring its generalizability and effectiveness across diverse patient populations and data sources. Additionally, the model’s design facilitates interpretability by allowing visual analysis of learned graph structures and channel attention mechanisms, providing valuable insights for clinical applications.

While Deepwalk-TS exhibits high accuracy and robust feature extraction capabilities, there are **several limitations to consider.** It is still affected by the inherent variability in individual physiological characteristics across different patients, which impacts the generalizability of the model to unseen data. Another limitation is the reliance on substantial labeled data, as the method's performance with raw, unsegmented signals is untested. Furthermore, while the visual interpretability of graph structures and channel attention is a strength, the framework lacks detailed mechanisms for clinical feedback loops.

Future research should focus on several key areas: Enhancing the robustness of the model to handle inter-individual variability via multi-view representation learning. To further enhance the model's performance across diverse datasets, we will explore

advanced techniques such as data augmentation, transfer learning, and domain adaptation. Additionally, techniques such as model pruning and unified representation will be employed to reduce computational complexity while preserving accuracy and interpretability. Focusing on these areas will not only enhance the performance and applicability of the method in epileptic seizure detection but also provide a foundation for researchers to extend its use to other neurological disorders and related fields. We have incorporated these discussions in the revised manuscript to provide a more balanced and comprehensive overview of the framework's strengths, weaknesses, and directions for future improvements.

3. What about the computational complexity of the proposed method? Some description related to computational complexity is required in the paper.

Thank you for raising the important point regarding the computational complexity of our proposed method. We included additional calculations in Experimental Results subsection to provide a more comprehensive analysis. The computational complexity of this model primarily stems from two key components: the Deepwalk-based GCN and the Guided-CNN Transformer.

Deepwalk-based GCN complexity can be approximated as $O(n \times k \times d)$, where n is the number of nodes ($n = 16$), k is the number of random walks per node ($k = 9$), and d is the dimensionality of the node embeddings. The random walk incurs higher costs with more nodes, walks, and embedding dimensions. Two-layer GCNs typically involve $O(v \times F \times F_1)$, where v is the number of nodes, F and F_1 are the original and output feature dimensions. The module's complexity is $O(n \times k \times d)+O(v \times F \times F_1)+ O(E \times F_1)$, E is the edges. Through extensive experiments, we have determined that the optimal number of EEG channels is 11, which helps manage the computational overhead. Guided-CNN Transformer complexity is focused on the self-attention mechanisms of Transformer. Q, K, V matrix shape is $n \times d$, $Q \times K_t = (n \times d \times n)$, scores $\times V = \text{softmax}(Q \times K_t / \sqrt{d}) \times V = O(n^2 d)$, so the complexity is $O(n^2 d + nd) = O(n^2)$, the quadratic complexity comes from the attention calculation between all input token pairs. Then, the outputs of the GCN space and Transformer time branches are fused, which mainly involves element-by-element operations or connections, which does not incur significant computational overhead. During training, the two branches are jointly optimized through techniques such as balanced loss function and mini-batch gradient descent, and the overall complexity is controllable up to $O(n^2)$. We have expanded the discussion of computational complexity in the revised paper, with a clearer breakdown of each component.

4. There is a huge amount of literature regarding EEG-derived fingerprints of seizure detection. Do authors consider that the incorporation of already described seizure-related features into their model could improve their results? Some discussion on that is needed. Without this more rigorous comparison with published work, this work is just another incremental addition.

Our work focuses on developing an end-to-end framework, the Deepwalk-TS, which automatically learns spatiotemporal features directly from raw EEG data. This approach eliminates the dependency on handcrafted features, allowing the model to

autonomously capture high-order spatial relationships and temporal dependencies. Below, we address the existing seizure features and works, results comparisons with existing research, and the incremental contributions of our proposed model.

In the revised manuscript, we have added a Related Works section, providing an overview of feature extraction and EEG-based seizure detection methods. In this paper, we focused on a deep learning-based approach that learns both spatial-temporal relationships between EEG channels, which is an area that has not been fully explored in the current works. Then, we revised the manuscript to clarify and expand on the integration of existing seizure-related features in the Experiment section. We conducted additional experiments incorporating mainstream handcrafted-based and GCN-based epilepsy detection methods, fusing features with representative methods like SSTFT and HGCN in Table 3. The results of these experiments have been updated in Table 3 of the revised manuscript.

We combined the optimal SSTFT features with our Deepwalk, achieving an accuracy of 99.01%, sensitivity of 98.95%, and specificity of 98.20%. This demonstrates that both our graph-based feature extraction and existing features contribute to improved epilepsy detection. Similarly, when we combined the representative HGCN method with our TS, we achieved an accuracy of 99.44%, marking a significant improvement over the original method. This proves that incorporating the described seizure-related features into their models enhances their performance. We acknowledge that integrating existing features could enhance the model's generalizability and robustness, providing complementary value and improving its practical efficacy.

While deep learning-based seizure detection is a growing research field, the novelty of Deepwalk-TS lies in its holistic integration of spatiotemporal features. Unlike existing methods that primarily focus on either spatial or temporal dimensions, our dual-branch architecture combines both, allowing the model to capture intricate relationships across EEG channels while effectively modeling temporal dependencies. This unified approach enhances the model's adaptability to complex and heterogeneous datasets, improving its robustness and accuracy in seizure detection. Therefore, our work should not be viewed as merely an incremental addition to existing research, but rather as a significant exploration in epileptic seizure detection.

5. The authors never state what level of performance would be considered adequate for a "real-world scenario" system that is useful and valid.

The reviewer's comment has inspired us to recognize the importance of defining performance thresholds for real-world epilepsy detection, which are crucial for assessing the practical applicability of our method. A system should achieve a minimum threshold of sensitivity (≥ 90%) is vital in clinical applications to reduce the risk of false negatives, which could lead to missed seizures and delayed interventions. In our experiments, the proposed Deepwalk-TS model achieves a sensitivity of 99.07%, which meets the necessity for ensuring timely detection of seizures. Meanwhile, high specificity (≥ 95%) is also important to avoid false alarms leading to unnecessary treatments or disruptions. Our model achieves a specificity of 98.97%,

which is suitable for distinguishing between seizure events and non-seizure events, minimizing the occurrence of false positives. In addition, the overall accuracy (≥ 95%) and Area Under the Curve (AUC ≥ 0.98) are also crucial for evaluating the overall performance of the detection system. Our model achieves an accuracy of 99.54% and an AUC of 99.06%, exceeding the necessary thresholds for precise epilepsy detection. In a real-world deployment beyond performance metrics, real-time detection with low-latency processing is essential for timely seizure detection and intervention. Although our model achieves high accuracy and sensitivity, further optimization is required for efficient real-time operation in portable EEG devices or closed-loop systems.

6. How the proposed emotion classification system be useful in the real-world scenario? Need to discuss.

We understand the question asks how our seizure detection work can be applied to emotion classification in real-world scenarios. While our research primarily focuses on EEG-based seizure detection, the core module of Deepwalk-TS can capture both spatial and temporal features from raw EEG signals and can be effectively applied to other domains, including emotion classification.

We have included a discussion on the application of the Deepwalk-TS algorithm for emotion classification in the revised version of the Discussion section. Adapting real-time EEG processing to detect emotional states offers vast potential across healthcare, human-computer interaction, education, and more. Emotion detection using EEGs can help track emotional distress, such as anxiety or depression, aiding clinicians in identifying early signs for timely intervention. It can also enhance customer interactions by detecting emotions like frustration or satisfaction. Additionally, seizure detection methods could extend to entertainment, such as video games, by tailoring user experiences based on emotional states. By extending our work into emotion classification, future research could create systems that respond dynamically to a person’s emotional state, offering better decision-making in various real-world scenarios. We have expanded on the potential applications of emotion classification using similar techniques in the revised manuscript.

Reviewer #1: The authors have performed a study on a deep-learning based model to detect epileptic seizures. The paper is well-written and the proposed methodology is robust. Some minor points need to be revised.

Thank you very much for your positive feedback and constructive comments. We greatly appreciate your recognition of the value of our work and the robustness of methodology. We have carefully considered your comments and incorporated the necessary revisions to further improve the clarity and depth of our paper.

1. Introduction: - page 1 the authors state that “early diagnosis of seizures is important for patients with refractory epilepsy to prevent irreversible damage to the patient’s brain”. This sentence makes too many assumptions in implying that single epileptic seizures can damage the brain irreversibly. Seizure detection is

an important goal, but this sentence needs to be reformulated.

We have revised the sentence and refined the logic of the introduction to ensure clarity and precision. The revised sentence now reads: "Early detection of epilepsy is crucial for timely intervention, reducing the risk of irreversible brain damage, and improving the overall quality of life for patients."

In this revised version, we emphasize the importance of early diagnosis in the high prevalence of epilepsy and the critical role it plays in preventing long-term damage and improving patient outcomes. We then segue into discussing the efficiency of EEG-based computer-aided detection systems and the challenges that remain, leading to the introduction of our proposed solution. This revision provides a clearer and more accurate context for our research.

2. Results: - Page 7 section 4.1 and 4.2 should be moved to Method section, since they do not represent a result of the current research.

We understand your concern regarding the placement of Datasets and Processing and Experimental Setup and Evaluation subsection. To address this, we updated the major section title from "Results" to "Experimental," which will more accurately reflect the content of these sections. We reorganized sections to improve the coherence and logical progression of the content. The revised experimental section includes datasets and processing, experimental setup and evaluation, experimental results, and ablation studies.

3. **Discussion: - Page 14 the authors have analyzed data from two different databases, one of them being represented by a pediatric population. This may influence the results and should be discussed.**

In the Discussion section, we initially overlooked the performance comparison across different datasets. In the revised version, we will address this gap by providing a detailed analysis. We evaluated our method using two distinct datasets: the pediatric patients at Boston Children's Hospital (CHB-MIT) and the neurological Siena dataset. The aim was to assess the robustness of the Deepwalk-TS model across diverse demographic groups, incorporating datasets that capture variations in age and physiological conditions influencing EEG signals. The Deepwalk-TS model achieved the best detection performance on both the CHB-MIT and Siena datasets, with accuracy of 99.54% and 99.18%, respectively. Our model is designed to handle such variability, enhancing its generalizability for practical clinical applications. We acknowledge that these demographic differences may affect the results. In the revised manuscript, we will provide a more detailed discussion of the potential limitations and advantages of using multiple datasets. All changes will be marked in blue for easy reference in the resubmitted version.

Reviewer #2:

Thank you very much for your detailed and insightful review of our manuscript. Your constructive feedback has been invaluable in helping us improve the clarity, depth, and overall quality of the paper. We have carefully addressed each of your

points and incorporated the necessary revisions into the manuscript, ensuring that it is more comprehensive.

1. **Please define SOTA in the abstract.**

To improve clarity, we have defined "SOTA" as "state-of-the-art" in the abstract, ensuring that readers understand the acronym. The revised abstract will explicitly mention that our proposed method achieves state-of-the-art performance in epileptic seizure detection. Additionally, we have corrected similar usage issues throughout the manuscript to ensure a more localized and accurate expression.

2. **Please mention the details in the abstract.**

We have expanded the abstract to provide a more comprehensive overview of the study. The revised abstract now includes additional details about our method, highlighting key components such as the the dual-branch architecture, and the spatiotemporal fusion approach. Additionally, we emphasize the key findings, including the model's performance metrics, such as accuracy, sensitivity, and specificity, in seizure detection. This revision ensures that readers gain a clearer understanding of both the methodology and the significant contributions of our work.

3. **Please add paper organization at the end of the introduction.**

In the Introduction section, we have added a "paper organization" subsection at the end. This section provides a brief overview of the manuscript's structure, guiding the reader through the key sections. This addition improves the clarity and flow of the paper, offering readers a clear road to the study.

The rest of this paper is organized as follows: The Related Work reviews existing research on epileptic seizure detection, focusing on various machine learning and deep learning approaches. Methodology Section describes the proposed Deepwalk-TS model, explaining its architecture, key components, and spatiotemporal features. The Experiments present the datasets used, performance metrics, and comparison with recent studies. The Discussion section analyzes the implications of the results, limitations, and future directions. Finally, the Conclusion section summarizes the main findings of the study and outlines the potential impact of the proposed approach in real-world epilepsy detection applications.

4. **The related work section is missing.**

The Related Work section was briefly covered in the Introduction, but we agree that a more detailed discussion would help clarify the context of our work. We have added a dedicated and comprehensive Related Work section in the revised manuscript. This section is divided into three main aspects: Traditional Feature-Based Methods, Deep Learning Methods, Recent Advances in Epileptic Detection. This subsection summarizes various methods that rely on handcrafted features and covers recent advances in deep-learning approaches for seizure detection. We highlight key research on integrating GCNs and Transformers into seizure detection. This enhanced section provides a clearer background and better situates our approach within the existing body of research.

5. **Fig.6, please indicate the blue and orange colours.**

We have updated the legend or title of Fig 6 to include a clear description of the blue and orange colors used in the figure. Fig 6 t-SNE visualization of the original and model-embedded distributions of the test data. Different colors represent different data feature types. The blue represents "normal" feature sample points, and the orange represents "epileptic seizure" feature sample points to ensure that it is clear to all readers. (a) Original data distribution. (b) Distribution of feature embeddings for model prediction results.

6. Limitations of the proposed system should be discussed in the paper.

We have added a discussion of the limitations of our proposed Deepwalk-TS in the revised manuscript. This includes potential challenges in real-world applications, as well as areas for future improvement and optimization. Our results demonstrate that Deepwalk-TS significantly outperforms traditional and contemporary models across multiple datasets. Despite these promising outcomes, the model's performance is affected by the inherent variability in individual physiological characteristics, which can impact the generalizability of new data. Additionally, the complexity of EEG sequences and high data dimensionality pose challenges in computational efficiency and scalability, and the model's dependency on training data quality is another limitation. While our method performs well in offline scenarios, real-time detection may present computational challenges.

Moreover, we propose several future research directions. These include refining the model's spatial exploration capabilities and enhancing robustness to inter-individual variability. It can optimize performance across diverse datasets through advanced data augmentation and transfer learning techniques. Additionally, we suggest reducing computational complexity while maintaining accuracy and interpretability for clinical deployment. These improvements will boost the Deepwalk-TS model's performance and applicability in epileptic seizure detection and lay the groundwork for its extension to other neurological disorders.

7. The learning curve (Accuracy and loss) of the DL models is missing from the paper.

Based on your valuable feedback, we have added learning curves, i.e., accuracy and loss curves, of the DeepWalk-TS model in the experiment. In the revised version, the curve will help readers better understand the training dynamics, convergence behavior, and overall performance of our model over time.

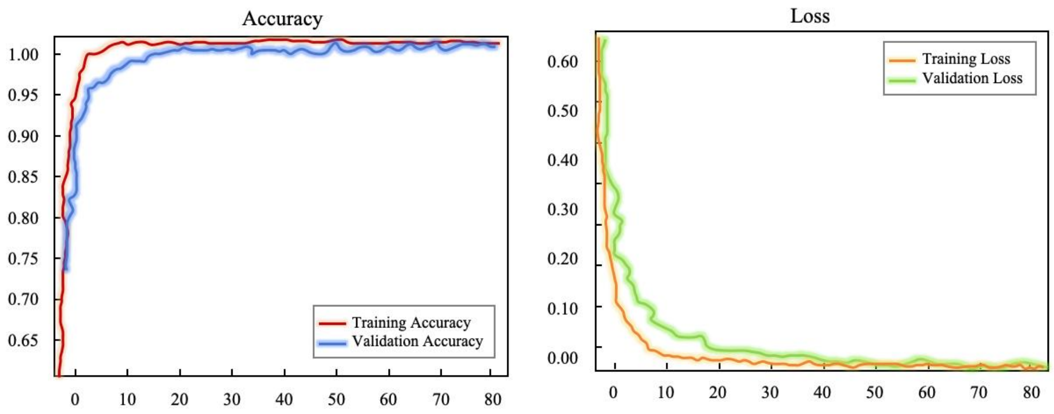

Fig 7. Accuracy and loss curves for DeepWalk-TS model during the training and validation process.

Reviewer #3:

We greatly appreciate the positive feedback regarding the potential and value of our work. Your detailed and insightful suggestions have been invaluable in helping us enhance the clarity and rigor of our paper. Below, we address each of the points you raised in your review.

1. The evaluation scores were significantly high with an accuracy of 99.54%. Why does it not reach 100%, and how can overfitting be understood in the proposed work? It is possible to add a description related to these concerns to the Discussion section, or wherever possible in the manuscript, it is feasible.

Our proposed Deepwalk-TS model has achieved 100% accuracy performance in the training phase and a very satisfactory 99.54% accuracy in the test phase. While 100% accuracy is rare in real-world tasks like seizure detection due to noise and variability in the data, the small gap reflects the differences between seizure-related and non-seizure signals. In clinical epilepsy monitoring, accuracy ≥ 95% is sufficient to alert doctors or instruments to make timely diagnostic interventions.

Overfitting occurs when the model memorizes noise or irrelevant patterns from the training data, reducing its ability to generalize to new data. The Deepwalk-TS model performs very well on the training data. To prevent overfitting, we evaluated the model on an independent test set with unseen samples. The high performance across multiple datasets shows that the model has good generalization ability. We have further elaborated on overfitting and validation strategies in the Discussion section to clarify the points raised.

2. Please mention the acronyms in full form when they are first used in the manuscript, such as "GMM," on page 9.

We have ensured that all acronyms are fully defined the first time they appear in the manuscript. Specifically, we have provided the full definition of "GMM" as a Gaussian Mixture Model in its first mention in the revised version. We also thoroughly reviewed the manuscript for similar instances and made necessary changes to enhance clarity and readability.

3. Please check Page no. 5, equation 2 and equation 3, the variables "bk" and "Dt" are defined somewhere or not.

We have improved the symbolic explanations of Eq. 2 and 3, and carefully checked the full text formula to ensure completeness and correctness.

$$\Pr(v_i \mid \phi(v_j)) = \prod_{k=1}^{[\log|V|]} \frac{1}{1+e^{-\phi(v_j)\cdot\varphi(b_k)}} \tag{2}$$

where $\varphi(b_k) \in R_d$ represents the parent node of tree node b_k. The path to v_j is identified by the sequence of tree nodes (b_0, b_1, … , log|v|), where b_0 = root and log |v|= v_j. We utilize the Huffman tree to assign shorter paths to frequently occurring vertices in deepwalk to speed up training time.

$$\mathbf{Z}_{ct}^{(l)} = \mathrm{Re}\,LU\left(\tilde{\mathbf{D}}_t^{-\frac{1}{2}}\tilde{\mathbf{G}}_t\tilde{\mathbf{D}}_t^{-\frac{1}{2}}\mathbf{Z}_{ct}^{(l-1)}\mathbf{W}_c^{(l)}\right) \tag{3}$$

where topology graph is (Gt, X), and feature graph is (Gf, X) that extract the shared information. $Z_{ct}^{(l)}$ is the node embedding of l layer, D is a diagonal matrix and represents the characteristic dimension of the node. $\tilde{D}_t$ represents the degree matrix of the vertex. $W_c^{(l)}$ is the l layer weight matrix of GCN and $Z_{ct}^{(l-1)}$ is the node embedding in the l-1 layer and $Z_{ct}^{(0)} = X$.

4. Also, check all the typos if they are available in the manuscript, such as in page no. 8 section 4.3.1, "...proposed model dem onstrated..."

We have carefully reviewed the manuscript for any other potential typos, including the one in section 4.3.1, the "…proposed model demo onstrated…" update to "…proposed model demonstrated…". We have thoroughly proofread the manuscript to correct grammatical errors, improve clarity, and enhance overall readability.

We greatly appreciate the positive feedback regarding the potential and value of our work. We are committed to addressing these concerns and will revise the manuscript to improve its clarity and completeness. We are excited to submit the revised version for you to look over. If you have any queries, please don't hesitate to contact me at the e-mail below.

Sincerely,

Xiaobing Deng
guoguo10201@163.com
