# Supplementary material for: A novel dual-branch network for comprehensive spatiotemporal information integration for EEG-based epileptic seizure detection
Source: PLoS One. 2025 Jun 26;20(6):e0321942. doi: 10.1371/journal.pone.0321942 (PMC12200712; doi:10.1371/journal.pone.0321942)
Supplement: Attachment — Submitted filename: Response_to_Reviewers_auresp_2.pdf [file pone.0321942.s004.pdf]

Response to Reviewers

Dear Editor and Reviewers:

We sincerely appreciate your letter and the reviewers' valuable feedback on our manuscript titled "A Novel Dual-Branch Network for Comprehensive Spatiotemporal Information Integration for EEG-based Epileptic Seizure Detection" (PONE-D-24-53519R1). We are grateful for the reviewers' positive comments, which have helped us further refine and improve our work. We have carefully reviewed all the suggestions and have made the necessary revisions in the Minor Revision resubmission. Below are our detailed point-by-point responses to each comment.

Editor Comments:

1. Please review your reference list to ensure that it is complete and correct.

We have carefully reviewed our reference list and thoroughly checked each citation to ensure completeness and accuracy. Missing fields, such as page numbers and other relevant details, have been corrected and supplemented where necessary. The revised manuscript now includes a fully verified and properly formatted reference list that adheres to the journal's guidelines.

2. While revising your submission, please upload your figure files to the Preflight Analysis and Conversion Engine (PACE) digital diagnostic tool.

Thank you for your valuable feedback, which has helped us improve the overall quality of our submission. We have carefully reviewed all figure files to ensure they meet the journal's formatting requirements. Please let us know if any further modifications are needed. We appreciate your guidance and support throughout the revision process.

Reviewer #1: The modifications have significantly enhanced the manuscript, and I find the current version to be well-structured and scientifically sound. I have no further major concerns at this stage.

Thank you for your positive feedback and recognition of our efforts in improving the manuscript. We sincerely appreciate your thoughtful review and valuable suggestions, which have helped us enhance the clarity, structure, and scientific rigor of our work. Your support and encouragement are greatly appreciated!

Reviewer #2: Please find the attached file for the comments. The paper needs minor revisions before the next submission.

1. The Fig.6 results show the overfitting issue. Please check it. The validation accuracy is higher than the training accuracy in the case of the SEED dataset.

We sincerely thank the reviewer for his insightful comments on the potential overfitting issue in the loss curve. Generally, a larger gap between the training and validation curves indicates strong overfitting, while a minimal gap indicates less severe overfitting. After carefully re-evaluating the training and validation process,

we found that the original validation curve was not fully plotted, which could have led to a misinterpretation of the results. We have corrected this issue in the revised figure and observe that the validation accuracy remains consistent with the training accuracy, indicating that the overfitting is small and does not significantly affect the overall model performance.

2. Please consider the following reference for your discussion

We sincerely appreciate the reviewer's suggestion to incorporate this reference into our discussion. The recommended paper offers valuable insights into EEG-based emotion recognition and the comparative performance of various supervised machine learning algorithms. We have now cited this reference in the revised manuscript, and we greatly appreciate the reviewer's insightful recommendation.

Reviewer #3: The author revised the manuscript where, all comments are addressed and responses are satisfactory. I do not have any further queries.

Thank you for your thoughtful review and positive feedback. We sincerely appreciate your time and effort in evaluating our manuscript. Your valuable comments have helped us improve the quality of our work, and we are grateful for your support!

We greatly appreciate the positive feedback regarding the potential and value of our work. We are committed to addressing these concerns and will revise the manuscript to improve its clarity and completeness. We are excited to submit the revised version for you to look over. If you have any queries, please don't hesitate to contact me at the e-mail below.

Sincerely,
Xiaobing Deng
guoguo10201@163.com
