# Supplementary material for: A novel dual-branch network for comprehensive spatiotemporal information integration for EEG-based epileptic seizure detection
Source: PLoS One. 2025 Jun 26;20(6):e0321942. doi: 10.1371/journal.pone.0321942 (PMC12200712; doi:10.1371/journal.pone.0321942)
Supplement: Attachment — Submitted filename: Comments R2.docx [file pone.0321942.s003.docx]

1. The Fig.6 results shows the overfitting issue. Please check it. The validation accuracy is higher than the training accuracy in the case of the SEED dataset.

Useful link:

- <https://stackoverflow.com/questions/43979449/higher-validation-accuracy-than-training-accurracy-using-tensorflow-and-keras>
- <https://www.quora.com/Can-validation-accuracy-be-higher-than-training-accuracy>

2. Please consider the following reference for your discussion:
   a. Electroencephalogram-Based Emotion Recognition: A Comparative Analysis of Supervised Machine Learning Algorithms, Data Science and Management, 2025, ISSN 2666-7649, <https://doi.org/10.1016/j.dsm.2024.12.004>.
